# Bioinformatics analysis and identification of upregulated tumor suppressor genes associated with suppressing colon cancer progression by curcumin treatment

**DOI:** 10.3389/fphar.2023.1218046

**Published:** 2023-09-05

**Authors:** Dan Wu, Zhenkai Fu, Wenna Liu, Yujia Zhao, Wenxuan Li, Qingqing Liu, Ying Liang

**Affiliations:** ^1^ Department of Pharmacy, Drug Development Center, Precision Pharmacy, Tangdu Hospital, Fourth Military Medical University, Xi’an, Shaanxi, China; ^2^ School of Basic Medical Sciences, Peking University, Beijing, China; ^3^ Department of Oncology, the First Affiliated Hospital, School of Medicine, Xi’an Jiaotong University, Xi’an, Shaanxi, China; ^4^ Laboratory of RNA Epigenetics, Institutes of Biomedical Sciences, Shanghai Medical College, Fudan University, Shanghai, China

**Keywords:** tumor suppressor genes, curcumin, mRNA high-throughput sequencing, molecular docking, colon cancer

## Abstract

Tumor suppressor genes (TSGs) are commonly downregulated in colon cancer and play a negative role in tumorigenesis and cancer progression by affecting genomic integrity, the cell cycle, and cell proliferation. Curcumin (CUR), a Chinese herb-derived phytochemical, exerts antitumor effects on colon cancer. However, it remains unclear whether CUR exerts its antitumor effects by reactivating TSGs in colon cancer. Here, we demonstrated that CUR inhibited HT29 and HCT116 proliferation and migration by cell-counting kit-8, colony-formation, and wound-healing assays. Furthermore, the comprehensive bioinformatics analysis of mRNA sequencing revealed that 3,505 genes were significantly upregulated in response to CUR in HCT116 cells. Kyoto Encyclopedia of Genes and Genomes and Gene Ontology analyses showed that the most upregulated genes were enriched in cancer pathways containing 37 TSGs. Five (*ARHGEF12*, *APAF1*, *VHL*, *CEBPA*, and *CASP8*) of the 37 upregulated TSGs were randomly selected for real-time fluorescence polymerase chain reaction and the verification results showed that these five genes were significantly reactivated after CUR treatment, suggesting that TSGs are related to CUR-mediated colon cancer inhibition. *ARHGEF12* is a newly identified TSG and a potential therapeutic target for colon cancer. Furthermore, molecular docking was performed to predict the binding sites of CUR and ARHGEF12, suggesting that CUR can prevent colon cancer cell invasion and metastasis by inhibiting ARHGEF12 and RhoA binding. In conclusion, the present study reveals that CUR inhibits colon cancer cell proliferation and migration by reactivating TSGs, revealing a new mechanism and potential target for colon cancer treatment.

## 1 Introduction

Colon cancer is a malignant tumor that is considered the third most common cancer worldwide with a high morbidity and mortality rate, thereby the fourth one to cause death (Selvam et al., 2019; Tie et al., 2022). Although great progress has been made in surgical techniques and treatments for colon cancer, the 5-year relative survival rate of patients with colon cancer has not changed markedly in the past decades (Shang et al., 2023). Currently, surgical treatment, chemotherapy, and radiotherapy are the major therapeutic modalities; however, these treatments usually have side effects that damage the immune system as well as liver and kidney functions and can even contribute to drug resistance (Zhang N. et al., 2021). Chinese herbal extracts have received increasing attention for their antitumor properties via diverse mechanisms in various types of cancer (Islam et al., 2022). For instance, baicalein, a novel toll-like receptor 4 (TLR4)-targeted therapeutic drug, inhibits the development of colorectal cancer (CRC) by inhibiting the TLR4/hypoxia-inducible factor-1/vascular endothelial growth factor signaling pathway (Chen et al., 2021). Astragaloside IV inhibits the development of hepatocellular carcinoma by persistently inhibiting fibrosis by regulating the pSmad3C/3L and nuclear factor erythroid 2-related factor 2/heme oxygenase-1 pathways (Zhang C. et al., 2021). Ginsenoside Rg3 effectively suppresses human CRC cell proliferation by inhibiting the transactivation of CCAAT/enhancer binding protein (C/EBP) and nuclear factor kappa-B (NF-κB), and the interaction of C/EBPβ with p65 (Yao and Guan, 2022). Ginsenoside Rh3 induces CRC cell apoptosis by upregulating the caspase-3 gene (Cong et al., 2020). Berberine inhibits the proliferation, migration and invasion of colon cancer cells by blocking the cell cycle in the G0/G1 phase through the Hedgehog signaling cascade (Sun et al., 2023). Ursolic acid suppresses colorectal cancer by downregulation of Wnt/β-catenin signaling pathway activity (Zhao et al., 2023). Although these herbal extracts exert important therapeutic effects on tumors, their antitumor mechanisms remain to be further explored. Therefore, there is an urgent need to identify additional Chinese herbal extracts and to better understand their molecular mechanisms that inhibit tumor development.

Curcumin (CUR) is one of the most common polyphenolic compounds extracted from the rhizomes of *Curcuma longa*. It is easily soluble in acetic acid, ketones, alkali, and chloroform, whereas it is insoluble in water at acidic and neutral pH. Owing to its hydrophobic properties, it can diffuse through cell membranes into the endoplasmic reticulum, mitochondria, and nucleus, where it can exert its action (Pricci et al., 2020). The therapeutic benefits of CUR have been demonstrated in multiple chronic diseases such as inflammation, arthritis, metabolic syndrome, liver disease, obesity, neurodegenerative diseases, and certain cancers (Aggarwal and Sung, 2009; Brockmueller et al., 2023). Recently, multiple studies have confirmed that CUR exerts strong anticancer effects against various types of cancer, such as breast, lung, hematological, gastric, colon, pancreatic, and hepatic cancers. The mechanisms involved include the inhibition of cell proliferation, induction of apoptosis, and suppression of cell migration and invasion via various molecular pathways (He et al., 2023; Zhang et al., 2023). CUR is a promising candidate as an effective anticancer drug that can be used alone or in combination with other drugs. It affects different signaling pathways and molecular targets involved in the development of several cancers. It has been reported that CUR plays an important role in anti-colon cancer. For instance, CUR inhibited 1,2-dimethylhydrazine-induced rat colon carcinogenesis and the growth of the *in vitro* cultured HT29 cell line by suppressing the peroxisome proliferator-activated receptor-γ signal transduction pathway; moreover, in human colon cancer HCT116 and HT29 cells, CUR induced the dissociation of hexokinase II (HKII) from mitochondria by downregulating the expression and activity of the HKII gene, leading to mitochondria-mediated apoptosis (Giordano and Tommonaro, 2019). In HCT116 cells, it has been reported that CUR increases miR‐491 expression, suppresses PEG10 expression, and consequently, silences the Wnt/β‐catenin signaling pathway as a mechanism of inducing apoptosis and inhibiting cell proliferation (Wu et al., 2020). Curcumin exerts its anticancer and antiproliferative activities by inducing senescence in colon cancer cells, and curcumin-induced senescence is accompanied by autophagy (Mosieniak et al., 2012). Curcumin regulates miR-21 expression and inhibits invasion and metastasis in colorectal cancer (Mudduluru et al., 2011). Curcumin inhibits colon cancer cell proliferation by targeting CDK2 (Lim et al., 2014). Based on the important clinical role of CUR in the treatment of colon cancer, there is an urgent need to investigate its anticancer mechanisms.

Tumor suppressor genes (TSGs) play opposing roles as oncogenes in the pathological process of cancer formation. It has been shown that TSGs play an antitumor role by affecting genomic integrity, the cell cycle, and cell proliferation. TSGs can be roughly classified into five groups based on their properties: (i) TSGs that promote cancer cells to enter into a certain stage of the cell cycle; (ii) TSGs that encode for effectors or ligands of signaling pathways that have inhibitory effects on cell proliferation; (iii) TSGs that encode for checkpoint-control proteins that initiate cell cycle arrest under conditions of DNA damage or chromosomal abnormalities; (iv) TSGs that encode for pro-apoptotic proteins; and (v) TSGs that encode for proteins that are involved in the repair of DNA damage (Gao et al., 2021; Gregory and Copple, 2023). According to existing studies, TSGs play an important role in inhibiting the occurrence and development of colon cancer. Sun et al. reported that 15-lipoxygenase-1, a TSG, promoted various antitumorigenic events, including cell differentiation and apoptosis, and inhibited chronic inflammation, angiogenesis, and metastasis, especially in colon cancer, and was downregulated in human colon polyps and cancers (Il Lee et al., 2011). Yang et al. reported that miR-1253 was a novel TSG in colon cancer that inhibited cell proliferation, migration, and invasion by targeting enhancer of zeste homolog 2 (Yang and Zhang, 2021). Cheng et al. reported that mindin acted as a TSG in a CRC mouse model via the mitogen-activated protein kinase/extracellular signal-regulated kinase signaling pathway, which directly suppressed colon cancer development (Cheng et al., 2020). Morin et al. reported that the inactivation of the adenomatous polyposis *coli* TSG initiated colonic neoplasia (Morin et al., 1997).

Based on the clinical importance of CUR in the treatment of colon cancer and its key role in colon cancer development, we explored whether CUR inhibited colon cancer by reactivating TSGs. Therefore, we verified the inhibitory effects of CUR on colon cancer by cell-counting kit-8 (CCK8), colony-formation, and wound-healing assays. The RNA sequencing (RNA-Seq) profiling of the control group and CUR-treated HCT116 cells revealed 3,505 upregulated genes. Furthermore, Kyoto Encyclopedia of Genes (KEGG) and Genomes and Gene Ontology (GO) analyses showed that among the upregulated genes, a total of 135 genes, including the largest number of differentially expressed genes (DEGs), were involved in cancer pathways. Besides, after the intersection of these 135 genes with the total TSGs downloaded from the TSGene database (https://bioinfo.uth.edu/TSGene/), we obtained 37 TSGs involved in cancer pathways that were upregulated in HCT116 cells by the action of CUR. We randomly selected five genes, *ARHGEF12*, *APAF1*, *VHL*, *CEBPA*, and *CASP8*, which were validated by quantitative polymerase chain reaction (qPCR) in HT29 and HCT116 cells. As expected, these five TSGs were significantly upregulated in both HT29 and HCT116 cells following CUR treatment. Furthermore, we predicted the binding site of ARHGEF12 and found that CUR might inhibit the invasion and migration of colon cancer cells by inhibiting the binding of ARHGEF12 to RhoA. Our results provide new insights into the use of CUR as a TSGs activator in colon cancer and suggest that TSGs play an important role in colon cancer.

## 2 Materials and methods

### 2.1 Compounds and reagents

Cur was obtained from MedChemExpress (#HY-N0005/CS-1490, purity ≥98%, CAS 458-37-7). Cell culture medium DMEM/HIGH GLUCOSE (4mML-Glutamine, 4500 mg/L Glucose) was purchased from CYTIVA (SH30022.01). CCK-8 was obtained from EnoGene Cell (E1CK-000208).

### 2.2 Cell lines and cell culture

Human colon cancer cell lines HT-29 and HCT-116 were obtained from the Cell Bank of the Chinese Academy of Science (Shanghai, China). All these cell lines were cultured in DMEM supplemented with 10% fetal bovine serum (FBS) and 1% penicillin-streptomycin under standard culture conditions (5% CO_2_, 37°C).

### 2.3 Cell proliferation assay

Cell proliferation was evaluated by the CCK8 assay. HT29 and HCT116 cells were seeded in 96-well plates at 1 × 10^4^ cells/well in a volume, and incubated overnight. Each cell line was then treated with 0, 5, 10, 20, 40 and 80 μM concentrations of CUR (dissolved in DMSO) for 24 h, the control group was treated with DMSO solvent corresponding to the experimental group. After treatment, 10 μL CCK8 reagent was added to a 96-well plate and incubated at 37 °C for 20 min, and then the absorbance was measured at a wavelength of 450 nm using an enzyme marker (M200Pro). IC50 values were obtained by nonlinear regression curve fitting analysis using GraphPad Prism 5.0.

### 2.4 Colony formation assay

Five thousand HT29 and HCT116 cells were inoculated in 6-well plates, respectively, and divided into control and CUR (30 μM) groups and cultured for 14 days until visible colonies appeared. The control group was treated with DMSO solvent of the same concentration as the experimental group. Then the colonies were stained with crystal violet for 20 min. The colony count was calculated using ImageJ software.

### 2.5 Cell migration assays

HT29 and HCT116 cells (80 × 10^4^ cells/well) were inoculated in 6-well plates and cultured for 24 h. CUR (30 μM) was added to the experimental group before scratching and a linear scratch was formed by quickly scratching over the monolayer cell surface using a 1 mL pipette. The control group was also replaced with a medium containing the same concentration of DMSO as the experimental group before scratches. Cell migration was observed under the microscope (EVOS XL Core) at 24 h and 48h, and wound healing was observed by comparing micrographs of the experimental and control groups at different times. The gap area was analyzed using ImageJ software.

### 2.6 RNA extraction and RT-qPCR analysis

HCT116 cells were treated with CUR (30 μM) for 24 h. Total RNA was then extracted from cells in the administered and unadministered groups, respectively, using Trizol reagent (Sangon Biotech, B511311-0100) and reverse transcribed with a reverse transcription kit (TaKaRa, Cat# RR047A). Quantitative RT-PCR, using the FastStart Essential DNA Green Master (Roche Diagnostics GmbH, Mannheim, Germany) was performed on Qtower2.2 equipment. Gene expression was calculated using the 2^−ΔΔCT^ method, with *GAPDH* as an internal references. Primers were synthesized as follows,

*GAPDH* forward: 5′-TGA​CTT​CAA​CAG​CGA​CAC​CCA-3′, and.

*GAPDH* reverse: 5′-CAC​CCT​GTT​GCT​GTA​GCC​AAA-3′; *ARHGEF12* forward: 5′-CTA​TCA​CCG​ACA​GAT​AGC​TCC​TCC-3′, and.

*ARHGEF12* reverse: 5′-CGC​TGA​ACA​AGA​CCA​TAT​ATC​TCG; *APAF1* forward: 5′-CAA​AGG​CTT​GGC​TCA​TGG​TTG​ACA-3′, and.

*APAF1* reverse: 5′-ATG​ATG​TAG​GAT​GTC​TTG​ATG​TCC-3′; *VHL* forward: 5′-CAG​CTA​CCG​AGT​CCT​CAT​GAC​T-3′, and.

*VHL* reverse: 5′-AGC​AGG​CAG​GTA​AGT​CAA​TTT​C-3′; *CEBPA* forward: 5′-CCG​GAT​CTC​GAG​GCT​TGC​CCG​A-3′, and.

*CEBPA* reverse: 5′-TCC​TCG​CAG​GGA​GAA​GCC​ACC​G-3′; *CASP8* forward: 5′-GGA​GCA​TCT​GCT​GTC​TGA​GCA​G-3′, and.

*CASP8* reverse: 5′-CAT​AAA​GAT​TTC​TGC​TGA​AGT​C-3′.

### 2.7 RNA preparation and RNA-seq

To investigate the effect of CUR on gene expression in colon cancer cells, HCT116 cells (30 × 10^4^ cells/well) were inoculated in 6-well plates and cultured for 24 h. The experiment was then divided into CUR (30 μM) treated and untreated groups for another 24 h. Total RNA was extracted using Trizol reagent for high-throughput sequencing. The RNA concentration and quality were determined using Nanodrop 2000 (Thermo Scientific) for library preparation. RNA-seq analysis was performed using Tophat2 (http://ccb.jhu.edu/software/tophat) to compare the sequencing reads to the human reference genome hg38. Reads were calculated using featureCounts (http://subread.sourceforge.net). Differentially expressed genes (DEGs) and statistical analyses were performed with DESeq2 (version 3.12) in R (version 4.0) (fold change >1.5, *p* < 0.05). Heat maps were created with Complex Heatmap (Bioconductor Project).

### 2.8 Molecular docking analysis

ARHGEF12–RhoA crystal complexes (PDB code:1X86) was obtained from the Protein Data Bank (http://www. rcsb. org/). Discovery Studio 2021 was used to perform molecular docking analysis. Before docking, CUR was prepared using “Prepare Ligands” module and CHARM force field was used for minimization, generating ten conformations. The protein was prepared using “Protein Preparation” module, allowing for the addition of hydrogen atoms and the deletion of unnecessary water. Subsequently, the proteins were optimized and minimized. Ligand binding sites are defined using the “Receptor-Ligand Pharmacophore Generation” module. The docking results were evaluated using hydrogen bond interactions and binding mode. The interaction 2D diagram of the CUR with residues of receptor was views in the “View Interaction” module. All structural figures were generated using PyMol 3.7.

### 2.9 Statistical analysis

All experiments were conducted in triplicate (n = 3) and the data are presented as the mean ± SEM. All statistical analyses were processed with GraphPad Prism 5.0. Differences of unpaired comparisons between two groups were analyzed using the ANOVA. *p*-value <0.05 was considered statistically significant.

## 3 Results

### 3.1 CUR inhibits the proliferation and migration of colon cancer cells

**FIGURE 1:**
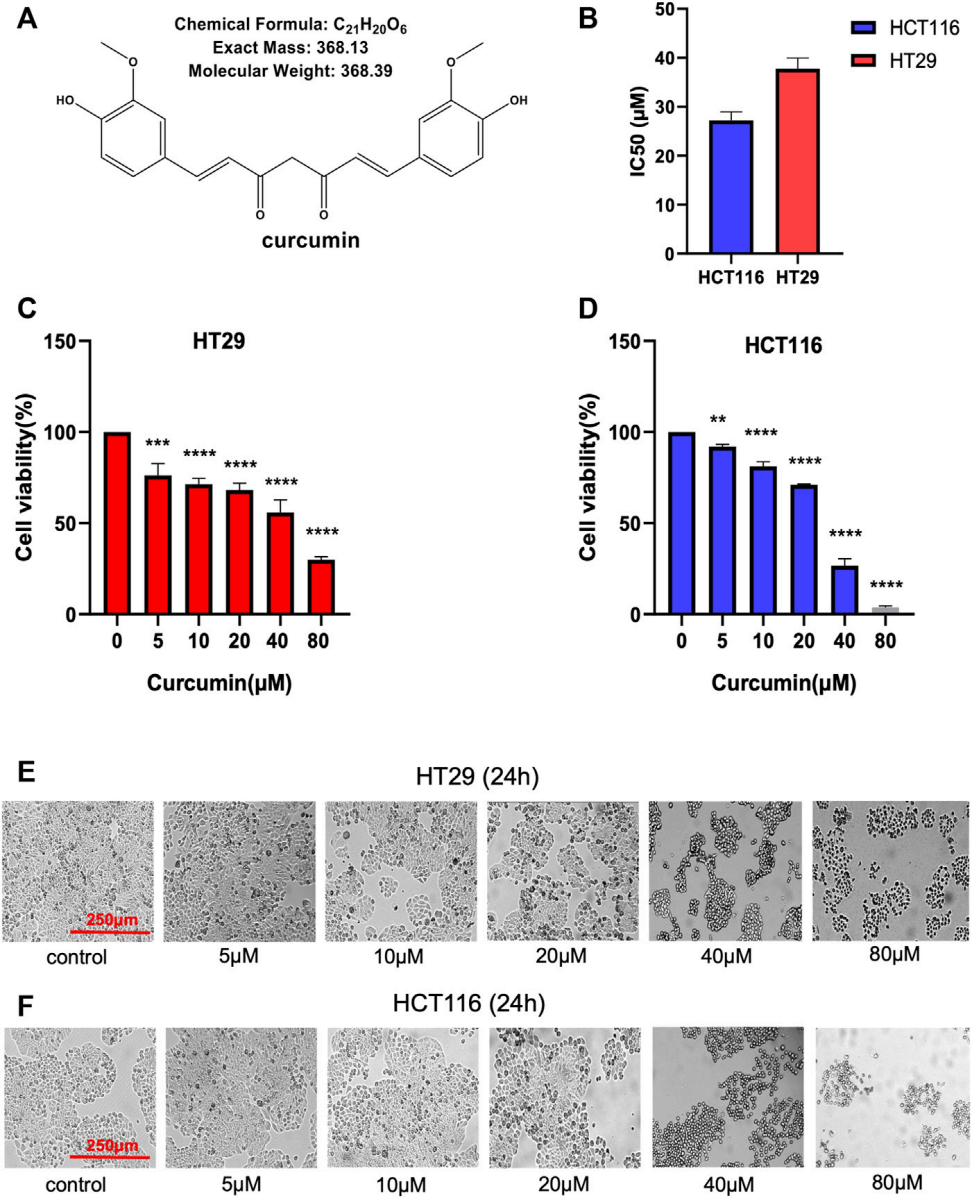
CUR inhibits the cell viability of HCT116 and HT29 cells in a dose-dependent manner. **(A)**. Chemical structure of CUR from ChemDraw. **(B)**. IC50 of CUR inhibition in colon cancer cells. **(C, D)**. The survival of HCT116 and HT29 cells was examined by CCK8 assay at 5μM, 10μM, 20μM, 40μM and 80 μM concentrations of CUR for 24 h. **(E, F)**. HCT116 and HT29 cell numbers and morphological changes were positively correlated with CUR concentrations. **** represents *p* < 0.0001, *** represents *p* < 0.001, ** represents *p* < 0.01.

Figure 1A shows the chemical structure of CUR. Firstly, we calculated the IC50 values of HCT116 and HT29 cells after CUR treatment by CCK8 assay. As a result, the corresponding IC50 at 24 h was 27.21 μM and 37.76 μM in HCT116 and HT29 cells, respectively (Figure 1B). Further, we investigated the effects of CUR on the viability of colon cancer cells. Cell viability was measured by CCK8 assay after treatment with CUR at concentrations of 5, 10, 20, 40 and 80 μM for 24 h. Figure 1C, D showed that CUR reduced cell proliferation in a dose-dependent manner. As the dose of CUR increased, the number of cells gradually decreased and the morphology shrank (Figure 1E, F).

**FIGURE 2:**
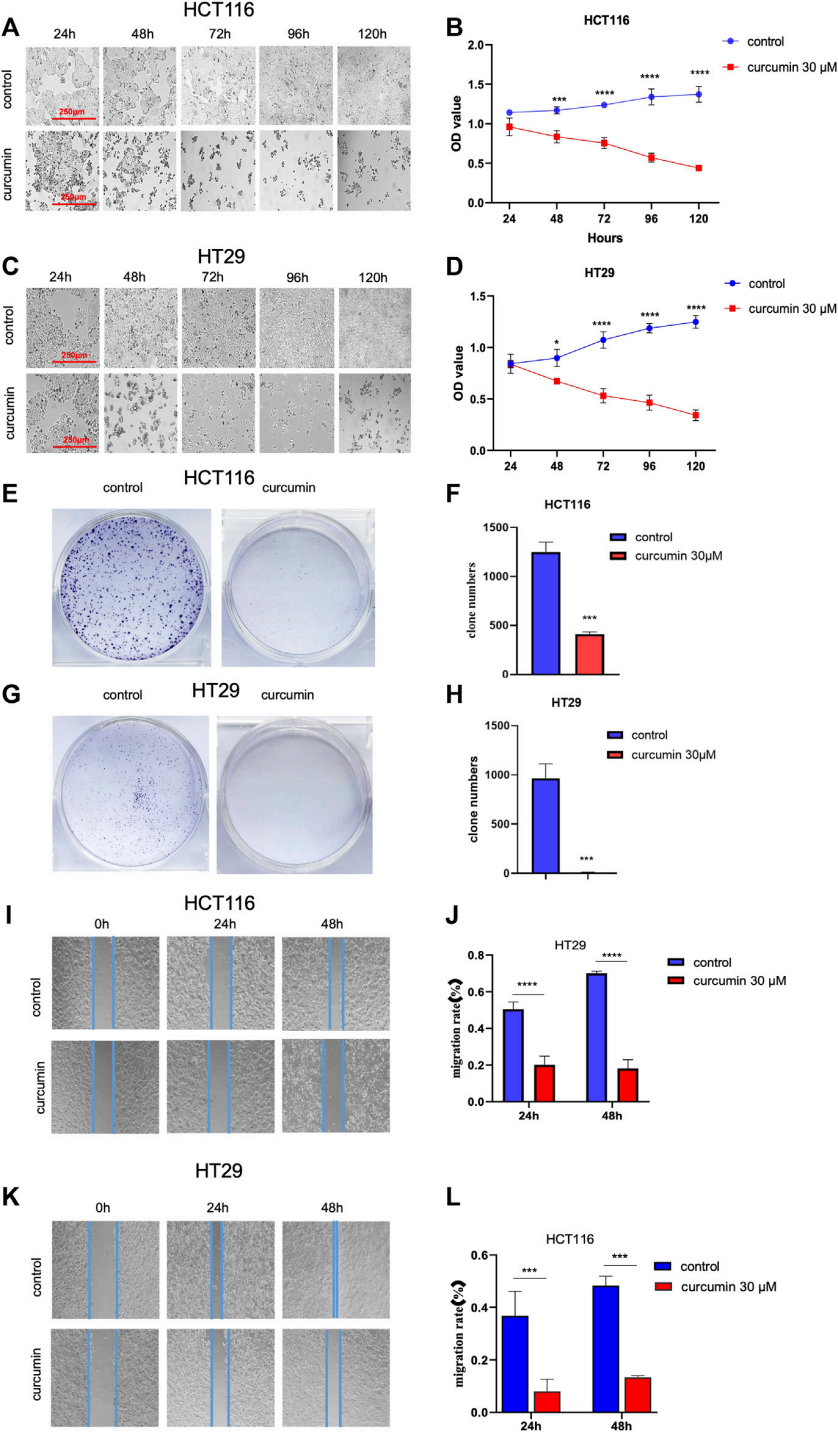
CUR inhibited the proliferation and migration abilities of HCT116 and HT29 cells in a time-dependent manner. **(A–D)**. The cell viability of 30 μM CUR was measured by CCK8 at 24h, 48h, 72h, 96 h and 120h, respectively. The viability and number of HCT116 and HT29 cells decreased in a time-dependent manner with CUR, and the cell morphology was also significantly wrinkled with the increase of drug action time. **(E–H)**. In colony formation assay, 30 μM CUR action for 24 h significantly reduced the number of clones formed by HCT116 and HT29 cells. **(I–L)**. 30 μM CUR significantly inhibited the migratory viability of HCT116 and HT29 cells at 24 h and 48 h, respectively. The cell migration pictures were gained by 200 times magnification under the microscope. **** represents *p* < 0.0001, *** represents *p* < 0.001, * represents *p* < 0.05.

Next, we used the CCK8 method to detect the effect of CUR on the proliferation of HCT116 and HT29 cells by CCK8 assay. The results in Figure 2A–D show that cell proliferation decreased in a time-dependent manner with increasing time of CUR treatment, and cell morphology was crinkled.Elucidation of the effects of CUR on colon cancer cell proliferation and migration by colony formation and wound healing assays. In the colony formation assay, the observations showed that the colony formation capacity of HCT116 and HT29 cells was reduced after treatment with CUR at 30 μM, indicated by significantly decreased colony numbers (Figure 2E-H). Wound healing assays showed that CUR treatment inhibited cell migration from 0 to 48 h (Figure 2I-L). The migration rates of the CUR treatment group in HCT116 and HT29 cells were 39.9% and 21.79% compared with those of the corresponding control group after CUR treatment for 24 h and 25.89% and 27.64% of the control group after CUR treatment for 48 h (*p* < 0.0001), respectively.

### 3.2 High-throughput sequencing identifies the upregulated tumor suppressor genes through the cancer pathway after CUR treatment

**FIGURE 3:**
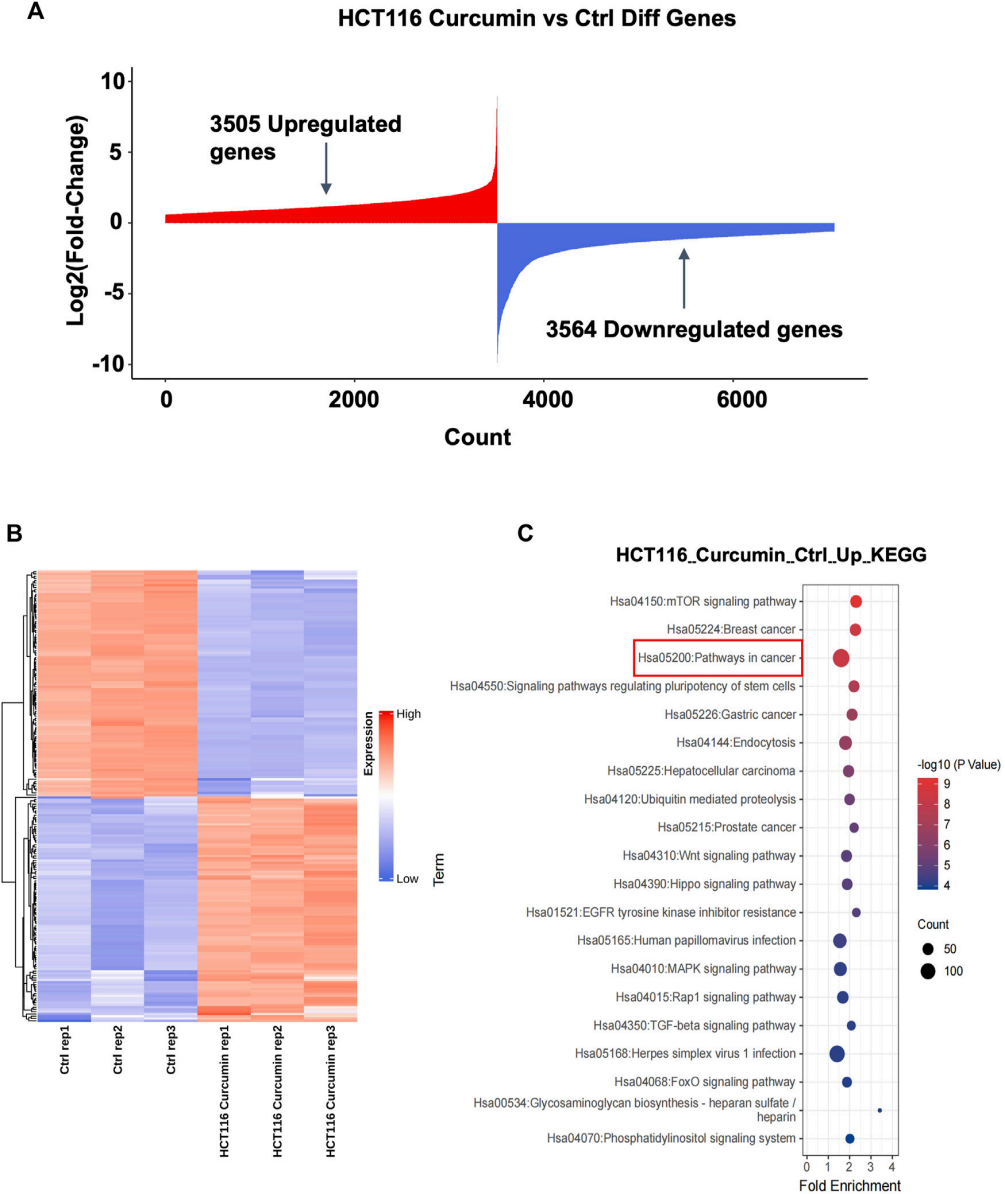
The highest number of genes involved in cancer pathways were upregulated by CUR. **(A)** A total of 3,505 upregulated genes and 3,564 downregulated genes were obtained by mRNA sequencing analysis under the effect of CUR. **(B).** Heat map of the top 100 differential genes. **(C).** KEGG enrichment analysis showed that the most upregulated of the differential genes were involved in the cancer pathway.

To investigate the molecular mechanism of CUR inhibition of colon cancer cell proliferation and migration, we treated HCT116 cells with CUR (30 μM) for 24 h, and collected HCT116 cells from the control and CUR groups separately for mRNA high-throughput sequencing analysis to determine the curcumin-related genes. The results are shown in Figure 3A, 3,505 genes were upregulated and 3,564 genes were downregulated. Cluster analysis by the top 100 differentially expressed genes in Figure 3B showed that these differentially expressed genes clustered significantly between the control and CUR group cell samples. KEGG pathway enrichment analysis showed that these differentially expressed genes after being acted upon by CUR were enriched in the cancer pathway in Figure 3C.

**FIGURE 4:**
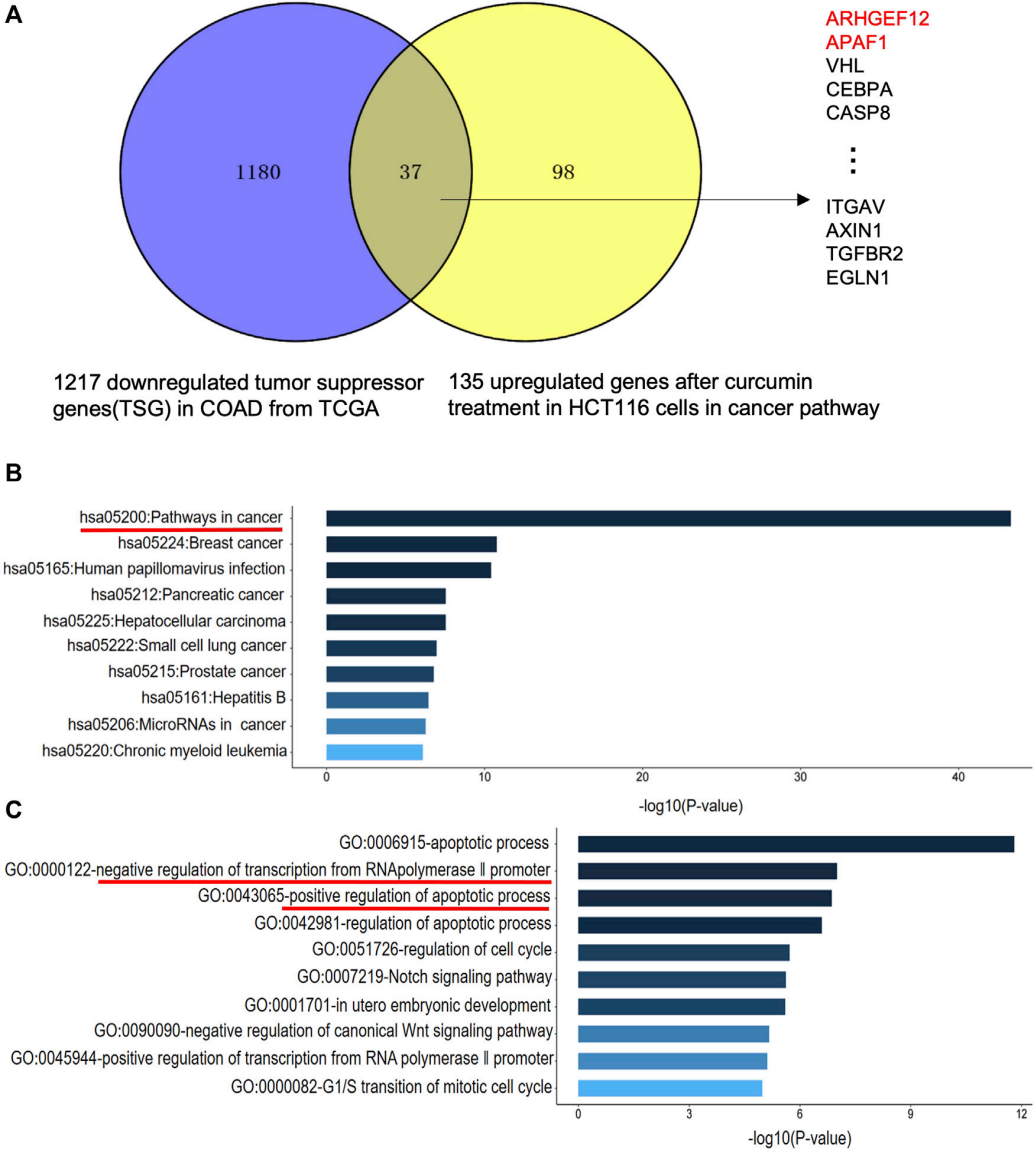
CUR may reactivate TSGs in COAD via the cancer pathway. **(A)**. CUR activated a total of 37 TSGs in the cancer pathway out of 135 upregulated genes. **(B)**. KEGG analysis of 37 upregulated TSGs treated with CUR. **(C)**. GO analysis of 37 upregulated TSGs after CUR treatment.

TSG is a key gene involved in DNA damage repair, suppression of cell mitosis, induction of apoptosis and prevention of metastasis. Hence, downregulation of TSG will lead to cancer development and progression. Therefore, re-upregulation of TSGs that are downregulated in cancers may prevent cancer progression (Wang et al., 2018). To explore whether CUR could reactivate the TSGs that were downregulated in colon cancer, we intersected the 1,217 tumor suppressor genes that were downregulated in colon cancer from TCGA database (https://bioinfo.uth.edu/TSGene/) with 135 genes that were upregulated in cancer pathways in HCT116 under the treatment of CUR, 37 TSGs that were reactivated under the action of CUR were obtained, including *ARHGEF12*, *APAF1*, *VHL*, *CEBPA*, *CASP8 et al* (Figure 4A). The results of KEGG and GO analysis are shown in Figure 4B, C. These 37 reactivated TSGs are mainly involved in the cancer pathways, which is consistent with the results of KEGG analysis of the first 100 upregulated genes in Figure 3C. In addition, GO profiling suggested that these 37 upregulated TSGs negatively regulated the transcription of RNA polymerase II promoter and positively regulated the apoptotic process. (Figure 4C), suggesting activation of these genes can suppress cell growth and proliferation.

### 3.3 CUR treatment reactivates the TSGs *ARHGEF12* and *APAF1* in colon cancer cells and correlated with a good prognosis for patients with colon cancer

**FIGURE 5:**
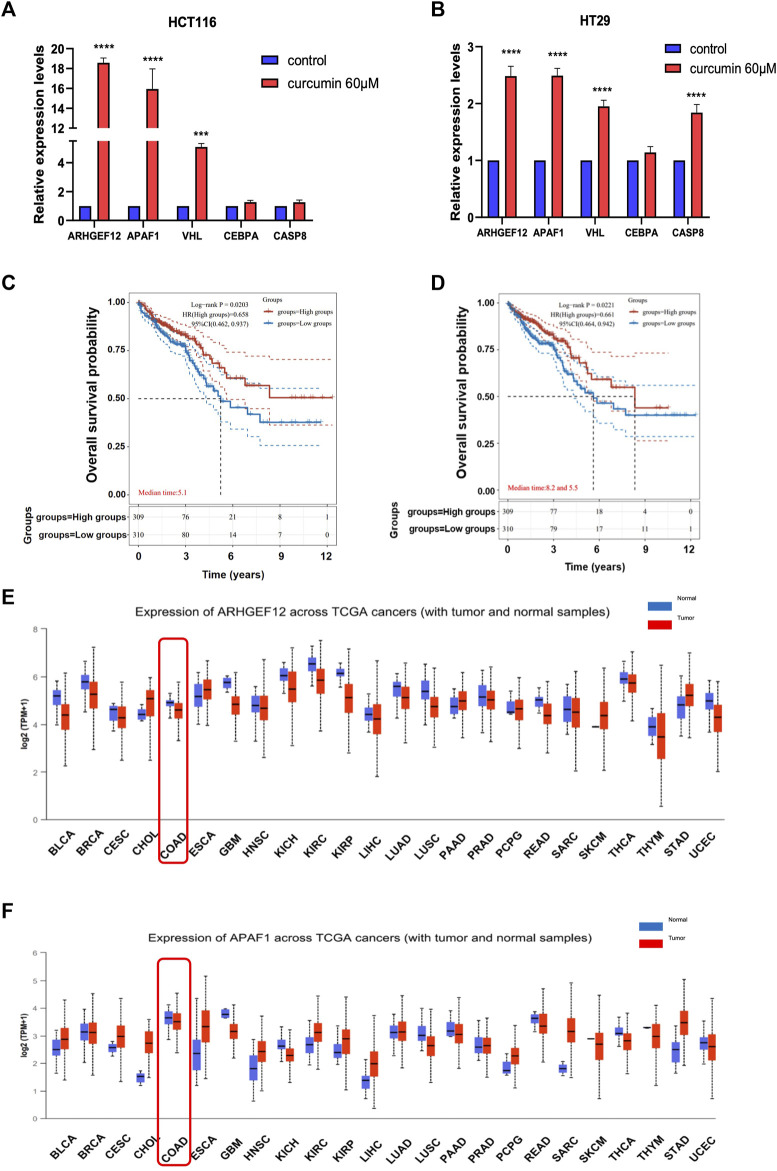
CUR increased TSGs *ARHGEF12* and *APAF1* in the cancer pathway, and these two TSGs are correlated with good patient prognosis in clinic practice. **(A, B)**. *ARHGEF12, APAF1, VHL, CEBPA* and *CASP8* were reactivated in HCT116 and HT29 cells after CUR treatment as verified by qPCR. **(C, D)**. Kaplan - Meier survival curve plots showed that activation of *ARHGEF12* and *APAF1* was positively correlated with the likelihood of patient survival time. **(E, F)**. pan-oncogene expression profiles of *ARHGEF12* and *APAF1*. **** represents *p* < 0.0001, *** represents *p* < 0.001.

To further verify that TSGs were indeed reactivated by CUR, we randomly selected five of the 37 upregulated TSGs: *ARHGEF12*, *APAF1*, *VHL*, *CEBPA*, and *CASP8* for qPCR assay. The results showed that the expression of these five TSGs was upregulated in both HCT116 and HT29 cells after CUR (60 μM) treatment, with significant differences in *ARHGEF12*, *APAF1*, *VHL* three genes in HCT116 and *ARHGEF12*, *APAF1*, *VHL*, *CASP8* four genes in HT29 (Figure 5A, B). Among them, *ARHGEF12* and *APAF1* genes, which were most significantly upregulated by CUR, were preferentially selected as candidate targets for CUR in colon cancer cells.

Considering that CUR can significantly reactivate TSGs *ARHGEF12* and *APAF1*, to further investigate whether upregulated *ARHGEF12* and *APAF1* expression in colon cancer correlates with patient prognosis, we analyzed colon cancer subtypes using Kaplan-Meier survival curves. We found in Figure 5C, D that high expression of *ARHGEF12* and *APAF1* was significantly correlated with a good prognosis for patients of colon cancer. Further, we queried the pan-oncogene expression profiles of *ARHGEF12* and *APAF1* in the TCGA (https://bioinfo.uth.edu/TSGene/) database using the UALCAN database (http://ualcan.path.uab.edu/), and as shown in Figure 5E, F, *ARHGEF12* and *APAF1* were significantly downregulated in colon cancer and many other types of cancers. In summary, both TSGs *ARHGEF12* and *APAF1* expression were downregulated in colon cancer and could be reactivated by CUR, and upregulated *ARHGEF12* and *APAF1* may be associated with favorable prognosis of patients.

### 3.4 Interaction of CUR with ARHGEF12–RhoA complex

Since ARHGEF12 has not been reported as a therapeutic target for colon cancer, we aimed to investigate the molecular mechanism by which CUR inhibits colon cancer by acting on ARHGEF12. Based on the reported researches, ARHGEF12 binds to RhoA to shape a functional complex, thereby enhancing cancer cell migration and invasion. (Lacoste et al., 2012; Ghanem et al., 2022). We speculate that CUR may block the binding of ARHGEF12-RhoA complex to suppress colon cancer cells, providing a new therapeutic strategy for colon cancer treatment.

**FIGURE 6:**
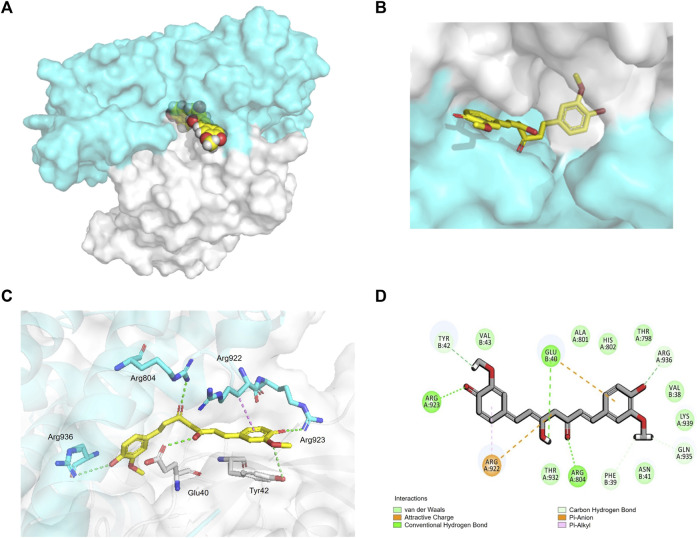
Interaction of CUR with ARHGEF12-RhoA complex. **(A, B)**. curcumin (yellow) occupied the cavity of the ARHGEF12-RhoA complex, ARHGEF12 (blue), RhoA (grey) (PDB code:1X86). **(C)**. Interactions of CUR with TYR 42 of RhoA, ARG923, ARG922, and ARG804 of ARHGEF12, respectively. **(D)**. Diagram of SantacruzaMate A interaction in the cavity formed by ARHGEF12-RhoA. The hydrogen bond is depicted as a green line, and the interaction of pi-alkyl is depicted as a purple line.

To verify whether CUR interacts with ARHGEF12–RhoA, we performed molecular docking to analyze its affinity and binding mode. In the structure of ARHGEF12–RhoA complexes, ARG 923 of ARHGEF12 formed multiple salt bridge interactions with ASP 45 and GLU 54 of RhoA, which was important for RhoA to be selected as a substrate (Aggarwal and Sung, 2009). In our study, we found that CUR occupied the cavity at the interface between ARHGEF12 and RhoA, indicating that CUR effectively inhibited interactions between ARHGEF12 and RhoA (Figure 6A, B). In the binding pocket, ARG 923 formed a direct hydrogen bond with the phenolic hydroxyl group of CUR at 2.8 Å. Additionally, ARG 804 of ARHGEF12 and GLU 40 of RhoA both formed stronger hydrogen bonds with CUR at 2.7 Å and 2.9 Å, respectively. ARG 936 of ARHGEF12 and TYR 42 of RhoA formed weaker hydrogen bonds with CUR at 4.0 Å and 3.7 Å. Moreover, ARG 922 of ARHGEF12 was packed in the hydrophobic core of CUR, which contributed to stabilizing the molecule at the binding site (Figure 6C, D). This suggests that CUR has a high affinity for ARHGEF12 and can effectively intervene in the interaction between ARHGEF12 and RhoA. Therefore, we hypothesized that CUR reactivated the *ARHGEF12* TSG to block colon cancer cell proliferation and migration via cancer pathways, whereas CUR might exert an inhibitory effect on invasion and migration by blocking the binding of ARHGEF12 to RhoA.

## 4 Discussion

Colon cancer ranks as the third most prevalent cancer worldwide, with an annual incidence of 1.1 million new cases. Furthermore, it is the second foremost contributor to cancer-related deaths, seriously threatening human health (Cervantes et al., 2023). The 5-year survival time has not been significantly improved by the current treatment methods; therefore, there is an urgent need to identify more treatment methods. Traditional Chinese medicine has been employed and widely acknowledged as an alternative approach in cancer treatment for centuries (Xiang et al., 2019). In particular, Chinese herbal medicine-derived phytochemicals, such as CUR, have demonstrated significant anti-tumor effects across various types of cancer (Miyazaki et al., 2023). CUR inhibits tumor development by reactivating TSGs. For instance, in lung cancer A549 and H460 cells, CUR significantly upregulates RARβ TSG expression at both the mRNA and protein levels (Jiang et al., 2015); CUR acts through the inhibition of DNA methyltransferases and the subsequent reactivation of *RASSF1A* in cancer, leading to its therapeutic effects (Dammann et al., 2017). However, it remains unknown whether CUR exerts its antitumor effects by reactivating TSGs in colon cancer.

**FIGURE 7:**
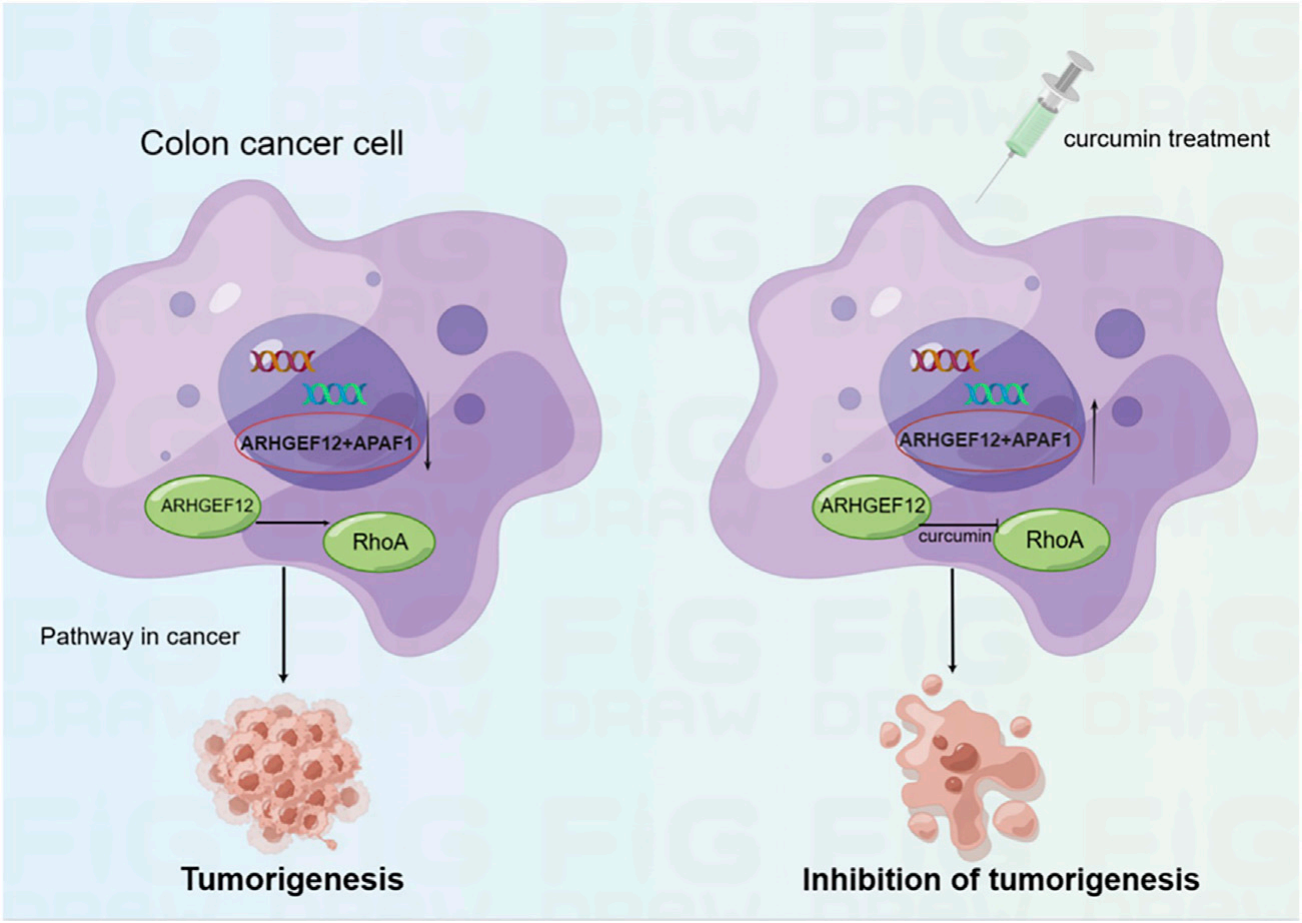
Schematic diagram showing CUR reactivates *ARHGEF12* and *APAF1* to block colon cancer cells proliferation and migration through cancer pathways. Moreover, CUR may inhibit the invasion and migration of colon cancer cells by blocking the binding of ARHGEF12 and RhoA.

In the present study, we performed mRNA-seq with CUR-treated or untreated colon cancer cell lines HCT116 and found 3,505 upregulated genes, among which 37 TSGs were significantly upregulated in cancer pathways, indicating that TSGs might play a critical role in colon cancer via cancer pathways. To confirm that these TSGs were indeed upregulated after CUR treatment, we selected 5 of the 37 upregulated TSGs for RT-qPCR. As expected, all five TSGs were upregulated, the most significantly upregulated TSGs were *ARHGEF12* and *APAF1* (Figure 5A, B). Furthermore, the upregulation of *ARHGEF12* and *APAF1* was found to be associated with a good prognosis in patients by Kaplan-Meier analysis (Figure 5C, D). Other researchers have also demonstrated the tumor suppressor role of these two TSGs in tumors. For example, the overexpression of *LARG* in breast and CRC cells demonstrated diminished cell proliferation and colony formation, as along with a significantly decreased cell migration rate in CRC cells, whereas *APAF1* played a key role in apoptosis and was significantly downregulated in colon cancer cells (Ong et al., 2009; Han et al., 2018). The present study provides groundbreaking evidence that CUR inhibited colon cancer development via the upregulation of the TSGs *ARHGEF12* and *APAF1.* Furthermore, we performed molecular docking and demonstrated that the curcumin can form a stable H-bond with ARHGEF12 and RhoA, respectively, indicating curcumin can bind tightly to these two proteins (Xiang et al., 2022; He et al., 2023), as shown in the schematic diagram (Figure 6, 7, by Figdraw). This, for the first time, suggested that CUR might play a role in inhibiting tumor invasion and migration by blocking the binding of ARHGEF12 and RhoA, which provided a theoretical basis for the CUR treatment of colon cancer.

## 5 Conclusion

We identified upregulated TSGs related to the inhibition of colon cancer progression after CUR treatment via comprehensive bioinformatics analysis and demonstrated that CUR inhibited the proliferation and migration of colon cancer cell lines by reactivating TSGs such as *ARHGEF12* and *APAF1* via cancer pathways. These TSGs are novel targets identified in the CUR-mediated inhibition of colon cancer and correlated with patient prognosis. Further, we predicted the binding sites of CUR and ARHGEF12 by molecular docking, suggesting that CUR may inhibit colon cancer invasion and migration by blocking the ARHGEF12-RhoA complex, which provides a theoretical basis for the molecular mechanism of CUR-mediated inhibition of colon cancer cells.

## Data Availability

The datasets presented in this study can be found in online repositories. The names of the repository/repositories and accession number(s) can be found in the article/Supplementary material.

## Abbreviations

CCK8, cell-counting kit-8; C/EBP, CCAAT/enhancer binding protein; CRC, Colorectal cancer; CUR, Curcumin; DEGs, Differentially expressed genes; FBS, Fetal bovine serum; GO, Gene ontology; HKII, Hexokinase II; KEGG, Kyoto Encyclopedia of Genes; NF-κB, Nuclear factor kappa-B; qPCR, Quantitative polymerase chain reaction; RNA-Seq, RNA sequencing; TLR4, Toll-like receptor 4; TSGs, Tumor suppressor genes.
